# The Effects of Cognitive‐Behavioral Consultation on Sexual Satisfaction and Quality of Life in Postmenopausal Women: A Randomized Clinical Trial

**DOI:** 10.1002/hsr2.71722

**Published:** 2026-01-06

**Authors:** Sousan Heydarpour, Sharare Khoshandam, Amir Jalali, Nader salari

**Affiliations:** ^1^ Department of Reproductive Health, School of Nursing and Midwifery Kermanshah University of Medical Sciences Kermanshah Iran; ^2^ Student Research Committee Kermanshah University of Medical Sciences Kermanshah Iran; ^3^ Substance Abuse prevention research center, Research, Institute for Health Kermanshah University of Medical Sciences Kermanshah Iran; ^4^ Department of Biostatistics, Faculty of Health Kermanshah University of Medical Sciences Kermanshah Iran

**Keywords:** cognitive‐behavioral consultation, menopausal, quality of life, sexual satisfaction

## Abstract

**Background and Aim:**

Ensuring a high quality of life during menopause is a critical aspect of women's health. This study aimed to evaluate the effects of cognitive‐behavioral consultation on sexual satisfaction and quality of life in postmenopausal women.

**Method:**

This randomized controlled trial included 70 postmenopausal women recruited from healthcare centers in Kermanshah, western Iran. Participants were randomly assigned to either an experimental group (*n* = 35) or a control group (*n* = 35) using a simple randomization method. The experimental group participated in 8 weekly sessions of group cognitive‐behavioral consultation, each lasting 70–90 min. Both groups completed the Menopause‐Specific Quality of Life and Women's Sexual Satisfaction Scale before and 1 month after the intervention. Recruitment took place between June and October 2018, and 70 participants completed the study. Data were analyzed using the Mann‐Whitney test, paired *t*‐test, and independent *t*‐test with SPSS (25), and significance was set at *p* < 0.05.

**Results:**

Before the intervention, no significant differences were observed between the groups in mean sexual satisfaction scores (*p* = 0.71) or quality of life mean scores (*p* = 0.128). However, post‐intervention, the experimental group demonstrated a statistically significant improvement in both sexual satisfaction (*p* = 0.001) and quality of life (*p* = 0.001) compared to the control group. No significant adverse events or side effects were reported.

**Conclusion:**

The findings suggest that group cognitive‐behavioral consultation can effectively improve sexual satisfaction and quality of life among postmenopausal women. These results underscore its potential as a valuable therapeutic approach to address challenges associated with menopause and enhance overall well‐being in this population.

**Trial Registration Clinical Trials:**

Iranian Registery of clinical trials‐Beta version, https://irct.behdasht.gov.ir/trial/14006. (**IRCT2017052814333N75**) registered (06‐04‐2017).

**Abbreviations**
KUMS: Kermanshah University of Medical Sciences
MENQOL: menopause‐specific quality of life
QOL: quality of life
SSSW: Women's sexual satisfaction scale

## 1 Introduction

Menopause is a natural part of the aging process in women, defined as the absence of menstrual periods for 12 consecutive months, marking the end of reproductive capability [1].

Declining estrogen levels during menopause, can include symptoms such as dizziness, heart palpitations, vaginal dryness, mood swings, sleep disturbances, headaches, joint and muscle pain, difficulty concentrating, and memory issues [1, 2]. The duration, severity, and impacts of these symptoms vary from one person or population to the next. Some women show severe symptoms that deeply impact their personal and social functioning as well as their quality of life [3].

Quality of life is an abstract concept that includes one's evaluation of their satisfaction with different aspects of life, including sexual, mental, social, and moral aspects [4]. Sexuality is one of the significant aspects of quality of life that can affect other life aspects, including physical, spiritual, and mental aspects [5]. Studies have found that more than half of postmenopausal women experience some form of sexual dysfunction [6, 7]. According to Vaz et al., study, vaginal dryness followed by painful sexual intercourse, and physical and mental problems brought about by menopause decreased the quality of life in women participating in the study [8].

Improvement in quality of life is pursued in all ages, and ascertaining the factors effective in decreasing quality of life and planning to promote the quality of life through psychological consultation and practical interventions has attracted attention [9].

Cognitive‐behavioral therapy (CBT), a form of psychotherapeutic intervention, helps individuals manage distressing and maladaptive thoughts that can adversely impact their quality of life [10].

Cognitive‐behavioral therapy represents a significant advancement in the field of psychological treatment and has garnered considerable interest among clinical professionals. The primary reason for this growing attention lies in the fact that, unlike other forms of behavioral therapy, CBT directly addresses individuals’ thoughts and emotions. This therapeutic approach assists patients in identifying and modifying distorted cognitive patterns and maladaptive behaviors through structured discussions and organized behavioral assignments [11].

Cognitive‐behavioral therapy aims to bring about changes in both cognitive and behavioral domains [12]. Several studies have investigated the effectiveness of CBT on women's sexual functioning. For example, Rostamkhani et al. conducted a study involving 45 postmenopausal women and found that those who participated in eight CBT sessions, each lasting 2 h, experienced significant improvements in their sexual schema compared to the control group [13]. Similarly, in a study of 198 women with sexual dysfunction, Babakhani et al. reported that four 2‐h sessions of CBT‐based counseling led to significant enhancements across all sexual functioning domains relative to the control group [14]. In another investigation, Kim et al. examined the efficacy of CBT for menopausal symptoms and quality of life in Korean perimenopausal women. Their findings indicated that participants in the CBT group showed notable improvements on both the menopausal symptoms and quality of life compared to those receiving treatment as usual [15].

Despite these promising findings, to the best of our knowledge, few studies have specifically examined the impact of CBT on sexual satisfaction and quality of life in postmenopausal women.

In light of this gap, the present study aimed to assess the effects of cognitive‐behavioral consultation on sexual satisfaction and quality of life among postmenopausal women attending healthcare centers in Kermanshah.

## 2 Methods

### 2.1 Design

In this controlled clinical randomized trial (1:1), 70 postmenopausal women visiting healthcare centers in Kermanshah were randomly selected and allocated to two groups, namely the experimental group (*n* = 35) and the control group (*n* = 35).

### 2.2 Sample Size Calculation

Sample size with 95% confidence (α‐1), 90% test power (β‐1), and considering the possibility of attrition, in each of the experiment and control groups were 35 participants. Ultimately, 70 postmenopausal women were enrolled in the study. Recruitment took place between June and October 2018.

### 2.3 Inclusion Criteria and Exclusion Criteria

The inclusion criteria were: Age range of 47–57, reaching natural menopause (in other words, they should have not taken drugs or had their ovaries removed using an operation to reach menopause), a minimum and maximum time interval of 1 year to 4 years since their last menstruations, living in Kermanshah, proficiency in Farsi, literacy, willingness to participate in the study.

The exclusion criteria were: Missing two or more consultation sessions, being addicted to drugs (both husband and wife), having any history of taking part in the consultation or educational programs, undergoing an incident or losing a first‐degree relative over last 3 months, suffering from dementia, heart diseases, lung diseases, metabolic diseases, and musculoskeletal disorders, using therapeutic hormones or any other drugs effective in decreasing menopausal symptoms, using psychedelic and psychiatric drugs, using therapeutic hormones, dealing with unpleasant incidents and facing any crisis in one's life disturbing participant's mental status over the past 3 months. Women and their husbands’ addiction to drugs.

### 2.4 Randomization

To select the health centers, Kermanshah City was divided into four regions—Northwest, Northeast, Southwest, and Southeast—based on geographical characteristics. A comprehensive list of health centers within each region was first compiled. Then, using a simple randomization method (random number table), one center from each region was selected.

Participants were subsequently selected through quota sampling, proportional to the number of postmenopausal women registered at each selected health center. For each selected center, a list of registered postmenopausal women was prepared, and individuals were randomly selected from these lists using a random number table.

Seventy identical cards were prepared, with 35 cards numbered as odd and 35 as even. The odd‐numbered cards were designated for Group A, while the even‐numbered cards corresponded to Group B. Each eligible participant randomly selected one card to determine their group assignment. Subsequently, the designation of Groups A and B as either the experimental or control group was determined randomly.

To ensure the integrity of the randomization process, participants were blinded to the group allocation method. Data collection was performed by a third party who was blinded to group assignments and trained specifically for this purpose.

### 2.5 Data Collection Tools

The data collection tools included a demographic form, Menopause‐Specific Quality of Life, and Women's Sexual Satisfaction Scale. These instruments were administered to two groups before and 1 month after the intervention.

#### 2.5.1 Demographic Form

This form included questions about the participants, such as age, spouse's age, education level, Job, residency, number of children, satisfaction with marital life, duration of marriage, Income, and BMI. The form was completed by participants in both groups before the intervention.

##### 2.5.1.1 Menopause Quality of Life (Menqol) Questionnaire

The Menopause Quality of Life (MENQOL) questionnaire consists of 29 items designed to assess women's quality of life across four domains: vasomotor (items 1–3), psychosocial (items 4–10), physical (items 11–25), and sexual (items 26–29). Each item is scored on a 7‐point Likert scale ranging from 0 to 6. A score of 0 indicates that the symptom was not experienced in the past month. A score of 1 indicates that the symptom was experienced but did not cause any distress. Scores of 2 through 6 reflect increasing levels of discomfort, with 2 representing a minor problem, 3 indicating some disturbance, 4 indicating a moderate disturbance, 5 reflecting relatively severe discomfort, and 6 denoting severe disturbance. According to the scoring guidelines, total scores between 0 and 58 suggest a good quality of life, scores between 59 and 116 indicate an average quality of life, and scores between 117 and 174 reflect a poor quality of life [16]. The MENQOL questionnaire was culturally adapted for use in Iran by Fallahzadeh et al. Its content validity was confirmed by experts, and its reliability was established with a correlation coefficient of 0.95 [17].

##### 2.5.1.2 Women's Sexual Satisfaction Scale (WSSS)

This scale is a self‐report, reliable, valid, and multidimensional tool that deals with women's anxiety and satisfaction. It was created in 2005 by Mostun and Tranpel. This scale consists of 30 questions, consisting of 5 dimensions, including Contentment (questions 1–6), Communication (questions 7–12), Compatibility (questions 13–19), Relational Concern (questions 19–24), and Personal Concern (questions 25–30). The scale of this scale is a 5‐point Likert (from strongly agree to strongly disagree). In this way, completely agree (1), slightly agree (2), neither agree nor disagree (3), slightly disagree (4), and completely disagree (5) are scored. Questions 1, 4, 5, 6, 9, 10, 11, 12 are scored in reverse [18].

Cronbach's alpha coefficients for the total score of the questionnaire in Iran were 0.96 and for its dimensions from 0.82 to 0.91, which were acceptable. Also, the test coefficients for the sexual satisfaction score and its dimensions in Iranian society were 0.73 to 0.97was obtained [19].

### 2.6 Intervention

The purpose of the sessions was explained to the participants by the second author during the first meeting, and informed written consent was obtained from them. Subsequently, both the intervention and control groups completed the demographic characteristics form, Women's Sexual Satisfaction Scale, and Menopause‐Specific Quality of Life Questionnaire. The control group received only routine care, including the assessment of body mass index (BMI), blood pressure, blood glucose, cholesterol, and triglyceride levels; screening for common female cancers; nutritional evaluation; colorectal cancer screening; and, when necessary, referrals to a physician, psychiatrist, or nutrition counselor.

The experimental group, in addition to routine care, received eight sessions (70–90 min) of group cognitive‐behavioral consultation. Counseling was done in groups of 11–12 participants (a total of three groups), by the second and third authors in the healthcare center in an appropriate place meeting the conditions required to provide consultation on a weekly basis.

Because the participants shared a common topic of study, were all the same sex, and could learn from each other's menopausal experiences, the group method was chosen. Furthermore, group therapy can help reduce irrational thoughts, anxiety, stress, and worries, which is a beneficial effect [20].

This study employed Rational Emotive Behavior Therapy (REBT), a type of cognitive behavioral therapy, to help clients by pinpointing and questioning unhelpful beliefs. The aim of REBT is to enhance emotional and behavioral well‐being. The method helps people swap harmful thoughts and actions for more sensible and constructive ones [21].

The topics covered in these eight sessions included: 1. Introduction of participants, establishment of session guidelines, and clarification of goals; 2. Education on menopausal issues, identification of participants’ concerns, and strategies to reduce anxiety and worry; 3. Exploration of participants’ irrational thoughts and beliefs related to menopause; 4‐5: Continued discussion on menopausal challenges; 6. Examination of participants’ thoughts, attitudes, and tendencies regarding menopause, including identification of negative beliefs and concerns;7. Emotional expression, training in self‐monitoring and stress‐reduction techniques; 8. Empowerment in the use of relaxation methods, session review, and conclusion. The detailed structure and content of these sessions are summarized in Table 1. To ensure the program was accurate, its content was reviewed by five clinical psychology experts, a nursing psychology PhD, and a psychiatrist.

**Table 1:** Contents of cognitive‐behavioral consultation sessions.

Session	Title and goal	Content	Applied techniques
First session	Familiarity with help‐seekers Making regulations of sessions Clarification of goals	− Welcoming − Implementing pre‐test − Introducing and − Introducing the members to each other and the counselor − Forming effective relationships with clients and expressing the goals − Emphasizing the confidentiality of the subjects proposed in the consultation sessions − Familiarizing members with the group consultation trend in general, and explaining the consultation structure (determining the time, place, and duration of sessions) − Evaluating the participants' information on menopause − Planning for next sessions − Providing pamphlets on menopause physiology in plain language	− Familiarization − Provision of information and informing − Techniques of forming effective relationships
Second session	Familiarization of groups members with menopause problems Determination of concerns and problems of the group members Decrease in their anxiety and concerns	− Reviewing menopause and its physical, mental symptoms − Expressing help‐seekers' problems during menopause − Discussing and challenging the problems of menopause by the members − Hearing help‐seekers' mental needs during menopause using targeted questions − Giving members some assignments concerning their problems during menopause − Writing down behavioral problems	Targeted questions
			Group discussion and challenges
			Catharsis
			Clarify
			Empathy
			Techniques of forming effective relationships
Third session	Familiarity of group members with their illogical thoughts and attitudes towards menopause	− Giving some assignments and group discussions − Discussing self‐image in terms of being menopausal, sexual activities, and marital life − Questioning and answering about menopause and related issues and marital status − Discussing irrational thoughts and emotional irresponsibility − Asking targeted questions about issues connected with menopause and quality of life − Expressing feelings and attitudes about self‐image and life concept	Targeted questions
			Group discussion and challenges
			Catharsis and clarity
			Empathy
			Techniques of forming effective relationships
Fourth and fifth sessions	Familiarity of group members with menopause problems	− Discussing physical, mental, and social problems in menopause − listening to help‐seekers' concerns and preoccupations with their expectations of themselves − Giving some assignments concerning physical, mental, social problems during menopause − Expressing their attitudes towards management of physical problems and psychological needs	Targeted questions and the use of third‐person questions
			Group discussion and challenge
			Catharsis
			Clarity and empathy
			Discovering thoughts and feelings
			Techniques of forming effective relationships
Sixth session	Familiarity with the thoughts, attitudes, and tendencies of the group members concerning the menopause problems Discovering negative concerns and thoughts of the group members	− Reviewing previous session contents and assignment of tasks by the members − Asking help‐seekers targeted questions and using of comparative method among choices − Expression of feelings about the understanding of one's sexual activities and the way to consider one's quality of life − Familiarizing with thoughts, emotions, and the extent of believing in negative beliefs during menopause − Recognizing participants' thoughts and feelings, and distinguishing negative beliefs from reality, and countering against one's negative beliefs − Giving some assignments to do at home − (Writing down negative thoughts and realities connected with these thoughts, writing down personal emotions)	Targeted questions
			Group discussion and challenge
			Catharsis
			Clarity
			Empathy
			Discovering thoughts and feelings
			Techniques of forming effective relationships
Seventh session	Discharge of group members' emotions Instructions in creating statistics and stress reduction methods	− Starting the session by reviewing previous sessions and assigning some assignments by the members and discussing them − Having help seekers express their feelings, fears, and attitudes − Speaking about the individual's thoughts and feelings and training in designing thoughts acceptance/rejection table − Discussing methods of communicating with one's spouse, family members and, relatives − Training in calming technique − Giving tasks on assertiveness and recommending doing calming practices at home	Targeted questions
			Group discussions and challenges
			Emotional catharsis, clarity
			Empathy
			Discovering thoughts and feelings
			Techniques of forming effective relationships
Eighth session	Empowerment of members to use calming methods Wrap‐up and closure	− Reviewing the program and goals again − Making sure of correct implementation of calming technique and assignments − Receiving feedback from group members on the successful and unsuccessful aspects of sessions and speaking about the thoughts and conclusions − Evaluating and following the sessions' programs after finishing the task − Reminding and necessary points − Wrap‐up and closure	Group discussions and challenges
			Clarity, empathy
			Playing roles
			Techniques of forming effective relationships

The intervention was delivered by the second and third authors.

**Figure 1:**
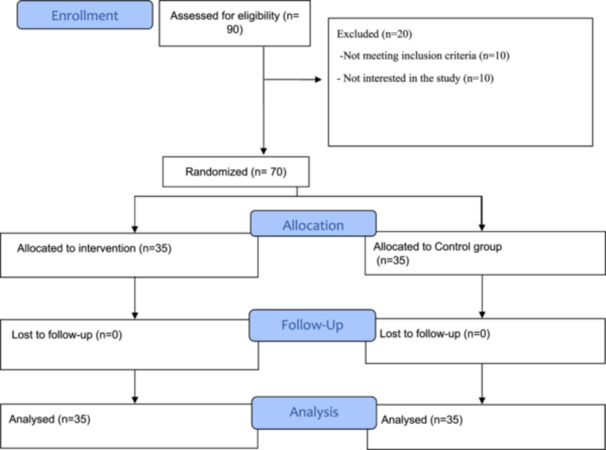
CONSORT flowchart of the study.

One month after the last group cognitive‐behavioral consultation session, the questionnaires were completed again by both groups (Figure 1).

### 2.7 Data Analysis

Data analysis was carried out using SPSS, Version 16, and descriptive statistics methods including frequency, Mean, Standard Deviation, and the analytic tests: chi‐square, paired *t*‐test, and independent *t*‐test. The significance level was considered less than 0.05 for all tests.

### 2.8 Ethics and Consent

The purpose of the study was explained to the participants by the second author during the first meeting. This study was performed in line with the principles of the Declaration of Helsinki. Approval was granted by the Ethics Committee of Kermanshah University of Medical Sciences (2017‐04‐19)/KUMS.REC.1396.6). Informed consent was obtained from all individual participants included in the study.

## 3 Results

We interviewed a total of 90 participants, of which 20 were eliminated because they did not meet the criteria for inclusion (*n* = 10) or declined to participate (*n* = 10). No participants were excluded during the follow‐up, and the final number of participants included in the statistical analysis was 70 (Figure 1).

The characteristics of the participants, along with the results of our comparative statistical analyses, are presented in Table 2. No significant differences were found in the individual characteristics among the two groups (*p* > 0.05) (Table 2).

**Table 2:** Individual characteristics of postmenopausal women in the experimental and control groups.

Variables		Experiment	Control	*p* value
		*N* = 35	*N* = 35	
		Mean ± SD	Mean ± SD	
		Or *N* (%)	Or *N* (%)	
Women's age (year)		50.3 ± 83.19	51.2 ± 57.75	0.26*
Spouse age (year)		57.5 ± 80.13	57.5 ± 23.86	0.70*
BMI^a^ (cm/m²)		27.3 ± 32.90	27.5 ± 31.33	0.82*
Duration of marriage		31.4 ± 91.71	31.3 ± 23.91	0.51**
Income	Less than 300$	22 (45.8)	26 (54.2)	0.22***
	300$ and more	13 (59.1)	9 (40.9)	
Education	Less than high school diploma	21 (51.2)	20 (48.8)	0.50***
	high school diploma and academics	14 (48.3)	15 (51.7)	
Job	Housekeeper	31 (50.8)	30 (49.2)	0.72****
	Working	4 (44.4)	5 (55.6)	
Residency	Personal	27 (50.9)	26 (49.1)	0.78***
	Rental	8 (47.1)	9 (52.9)	
Number of children	Less than three children	25 (50)	25 (50)	> 0.99***
	Three children and more	10 (50)	10 (50)	
Satisfaction with marital life	High	9 (50)	9 (50)	0.66*****
	Low	17 (45.9)	20 (54.1)	
	To some extent	9 (60)	6 (40)	

* Mann–Whitney U test

^a^ Body Mass Index.

** Independent *t*‐test.

*** Yates' correction.

**** Fisher's exact test.

***** Chi‐square.

Kolmogorov‐Smirnov test showed that the variable of quality of life and its four subscales, and also the sexual satisfaction variable and its five subscales, before and 1 month after the intervention, were normally distributed in the control group and experiment group. Therefore, the parametric test was used to analyze them.

As presented in Table 3, the independent *t*‐test showed there was no statistically significant difference (*p* = 0.128) between the quality of life in the control and experimental groups before the intervention. However, there was a significant difference (*p* < 0.05) between the two groups in terms of quality of life 1 month after the intervention.

**Table 3:** Comparison between the mean quality‐of‐life scores of the control and experimental groups before and 1 month after the intervention.

Variables/groups	Before	One month after	*p* value
	Mean ± SD	Mean ± SD	
Vasomotor			
Experiment	11.89 ± 3.46	7.97 ± 2.17	0.001*
Control	10.46 ± 4.5	12.17 ± 3.73	0.002*
*p* value	0.142 **	0.001 **	
Psychosocial			
Experiment	19.80 ± 5. 56	11.43 ± 3.35	0.001*
Control	18.09 ± 4.45	16.34 ± 5.17	0.015*
*p* value	0.159**	0.001**	
Physical			
Experiment	40.54 ± 0. 4	30.6 ± 5.67	0.001*
Control	40.8 ± 7.34	37.03 ± 8.59	0.005*
*p* value	0.856 **	0.001**	
Sexual			
Experiment	11.49 ± 4.08	5.89 ± 1.64	0.001*
Control	10.23 ± 3.45	10.17 ± 3.32	0.624*
*p* value	0.169**	0.001**	
Quality of life total			
Experiment	83.71 ± 10.11	55.89 ± 8.03	0.001*
Control	79.57 ± 12.28	75.71 ± 11.51	0.017*
*p* value	0.128**	0.001**	

* Paired *t*‐test.

** Independent *t*‐test.

The independent *t*‐test showed that there was no significant difference (*p* > 0.05) between the four subscales of quality of life in the control and experimental groups before the intervention. However, the difference (*p* < 0.05) was statistically significant 1 month after the intervention.

Paired *t*‐test showed that there was a statistically significant difference (*p* < 0.05) in the four sub‐scales of quality of life before and 1 month after the intervention in the experimental group. Paired *t*‐test also showed in the control group that there was a statistically significant difference (*p* < 0.05) among vasomotor, psychosocial, and physical subscales before and 1 month after the intervention.

As presented in Table 4, the results of the paired *t*‐test showed that there was a significant difference (*p* = 0.001) between the average total score of sexual satisfaction before and after the intervention in the experimental group. However, there was no significant difference (*p* = 0.574) between the mean score of sexual satisfaction before and after the intervention in the control group.

**Table 4:** Comparison of the Mean and SD of the total score and subscales of sexual satisfaction between the experimental and control groups.

Variables/groups	Before	1 month after	*p* value
	Mean ± SD^a^	Mean ± SD	
Satisfaction			
Experiment	14.57 ± 1.39	15.31 ± 2.28	0.091*
Control	14.91 ± 1.12	14.89 ± 1.65	0.405*
*p* value	0.258**	0.0001**	
Relationship			
Experiment	17.54 ± 1.69	18.26 ± 2.89	0.091*
Control	16.57 ± 3.01	16.91 ± 3.33	0.228*
p‐value	0.076**	0.69**	
Adjustment			
Experiment	22.97 ± 3.54	29.11 ± 2.37	0.001*
Control	22.69 ± 2.43	22.86 ± 3.67	0.769*
*p* value	0.001**	0.69**	
Relationship anxiety			
Experiment	19.43 ± 3.42	25.51 ± 2.11	0.001*
Control	20.11 ± 2.83	19.83 ± 2.67	0.456*
*p* value	0.001**	0.363**	
Personal anxiety			
Experiment	22.77 ± 2.84	26.46 ± 1.67	0.001*
Control	22.37 ± 2.85	22.77 ± 3.3	0.442*
*p* value	0.001**	0.588**	
Sexual satisfaction total			
Experiment	97.29 ± 8.1	114.66 ± 5.82	0.001*
Control	96.66 ± 5.77	97.29 ± 8.58	0.574*
*p* value	0.001**	0.71**	

* Paired *t*‐test.

** Independent *t*‐test.

^a^ Standard Deviation.

Moreover, the results of the independent *t*‐test showed that there was no statistically significant difference (*p* = 0.71) between the mean score of sexual satisfaction in both the experimental and control groups before the intervention. However, the difference was significant (*p* = 0.001) in both the experimental and control groups after the intervention.

Independent *t*‐test showed that there was no statistically significant difference (*p* > 0.05) among the three subscales, namely compatibility, relational concern, and personal concern, of the questionnaire on women's sexual satisfaction in the control and experimental groups before the intervention. However, the difference was statistically significant (*p* < 0.05) 1 month after the intervention. There was no significant difference (*p* > 0.05) between the two subscales of contentment and communication before and 1 month after intervention in the experiment and control group.

Paired *t*‐test showed that there was a statistically significant difference (*p* < 0.05) between four subscales of communication, compatibility, relational concern, and personal concern before and 1 month after the intervention in the experimental group. However, there was no statistically significant difference (*p* = 0.091) in the satisfaction subscale before and 1 month after the intervention in the experimental group. Moreover, the paired *t*‐test showed in the control group that there was no statistically significant difference (*p* > 0.05) between the five subscales of contentment, communication, compatibility, relational concern, and personal concern before and 1 month after the intervention. No significant adverse events or side effects were reported.

## 4 Discussion

The present study investigated the effect of cognitive‐behavioral consultation on menopausal women's quality of life and sexual satisfaction. The findings indicated that holding group consultation sessions with a cognitive‐behavioral approach promoted menopausal women's quality of life in the experimental group. There was no significant difference in the quality of life and its dimensions between the control and experimental groups before the intervention; however,

the difference was significant 1 month after the intervention. In this regard, Parsa et al. also found in their study that providing supportive consultation to women during menopause can promote their quality of life as well as their physical and mental health [22].

Moilanen et al. also concluded in their study that the improvement in menopausal women's satisfaction with life will be possible following the increase in their awareness of menopause status, physical activities, awareness, and control over their weight [23].

A study by Rathnayake et al. also found that teaching hygiene tips to enhance menopausal women's awareness and knowledge about the correct lifestyle in this course has improved their quality of life as well as their health [24].

Furthermore, according to the findings of the current study, group consultation with a cognitive‐behavioral approach improved four aspects of quality of life, including vasomotor, mental‐social, physical, and sexual aspects. Kim et al. ascertained that the healthcare instructions provided by the nurses to the patients can promote quality of life and its subscales, including emotional, social, and physical functioning [25].

In the study by Parsa et al. the cognitive consultation also improved help seekers’ scores after the intervention in different aspects, including vasomotor, physical, social, and sexual aspects [22].

In the study by Farhodimoghadam et al. cognitive‐behavioral counseling improves the lifestyle of pregnant women [26].

It appears that the positive impact of cognitive‐behavioral consultation on the quality of life of menopausal women stems from its role in helping them identify and replace negative thoughts related to menopause with more positive ones. This type of consultation is also conducted to reduce harmful behaviors that negatively affect quality of life. Consequently, through changes in thought patterns, behaviors, and emotional responses, women experience a significant improvement in their well‐being and overall quality of life.

Furthermore, the findings of the present study indicated that conducting group consultation sessions using a cognitive‐behavioral approach led to a significant improvement in sexual satisfaction within the experimental group. In contrast, no significant changes were observed in the control group. A statistically significant difference in sexual satisfaction was also identified between the experimental and control groups 1 month following the cognitive‐behavioral intervention. Supporting these findings, Öztürk and Arkar (2017) reported that cognitive‐behavioral therapy enhanced sexual satisfaction and functioning in couples experiencing vaginismus [27].

Similarly, Mofaraheh et al. found that consultation improved not only sexual satisfaction among couples but also their awareness and understanding of sexual issues [28].

Parsa et al. demonstrated that cognitive‐behavioral consultation enhanced both sexual satisfaction and the quality of sexual activity in postmenopausal women [22].

Zamani et al. also concluded that sexual health consultation positively influenced sexual satisfaction and sexual functioning in couples [29].

These findings may be explained by the generally limited and often inaccurate sexual knowledge within the cultural context of Iranian men and women, particularly among women. Their access to reliable information about sexual functioning is restricted, and prevailing beliefs, attitudes, and cognitive distortions surrounding sexual matters frequently shape their perceptions. Such misconceptions and irrational thoughts can adversely affect sexual functioning, especially in women [25].

The observed improvement in women's sexual satisfaction may be attributed to the cognitive‐behavioral intervention's focus on restructuring irrational beliefs regarding oneself, one's partner, or the relationship. Techniques such as positive self‐talk, attentional control, and assertive communication, which are integral components of cognitive‐behavioral therapy, can effectively address and alleviate cognitive aspects of sexual desire disorders in women. In addition, behavioral practices prescribed by cognitive‐behavioral consultation in the experimental group were not limited to mechanical and physical factors, but these practices could lead to appropriate behavioral reactions by group members, thereby improving their performance and preventing unwanted stresses and anxiety. The internal conflicts of experimental group members will be naturally affected and improved by these behavioral practices. This is the case because the consultation allows the individuals involved in the intervention to express more of their sexual emotions, and speak more freely about their interests and wants, thereby mitigating many ambiguous issues and wrong perceptions about menopause in this group of women [30]. This study possesses several methodological strengths. First, there was no sample attrition during either the intervention period or the 1‐month follow‐up, which bolsters the statistical validity of the findings. Second, the random allocation of participants to the experimental and control groups reduced the risk of selection bias. Additionally, the use of a specialized and validated questionnaire tailored to assess the quality of life in menopausal women strengthened the relevance and accuracy of the outcome measures. Nonetheless, a primary limitation of the study was the inability to blind participants and researchers, which may have introduced performance or assessment bias.

Another limitation of the study is the short follow‐up period (only 1 month), which may not be sufficient to assess the sustainability of the intervention's effects. Additionally, the absence of an evaluation of potential mediating variables (e.g., body image, cognitive schemas related to sexuality) represents another limitation of the research.

## 5 Conclusion

Cognitive‐behavioral consultation improves menopausal women's quality of life. Therefore, it is recommended that this type of consultation be used alongside other services to promote quality of life in menopausal women. Further studies with 6‐month to 1‐year follow‐up are recommended. Moreover, further studies are required to investigate the generalizability and cost‐effectiveness of the intervention.

## Author Contributions

**Sousan Heydarpour:** conceptualization, methodology, supervision, project administration, writing – review and editing. **Sharare Khoshandam:** writing – original draft, resources, methodology. **Amir Jalali:** validation, methodology. **Nader salari:** methodology, data curation, formal analysis, validation.

## Consent

The authors have nothing to report.

## Conflicts of Interest

The authors declare no conflicts of interest.

## Transparency Statement

The lead author Sousan Heydarpour affirms that this manuscript is an honest, accurate, and transparent account of the study being reported; that no important aspects of the study have been omitted; and that any discrepancies from the study as planned (and, if relevant, registered) have been explained.

## Data Availability

The data that support the findings of this study are available from the corresponding author upon reasonable request.
